# Metformin alleviates inflammation through suppressing FASN-dependent palmitoylation of Akt

**DOI:** 10.1038/s41419-021-04235-0

**Published:** 2021-10-12

**Authors:** Wenfang Xiong, Kuo-Yang Sun, Yan Zhu, Xiaoqi Zhang, Yi-Hua Zhou, Xiaoping Zou

**Affiliations:** 1 Department of Gastroenterology, Nanjing Drum Tower Hospital, The Affiliated Hospital of Nanjing University Medical School, 321 Zhongshan Road, Nanjing, 210008 Jiangsu PR China; 2 State Key Laboratory of Pharmaceutical Biotechnology, Department of Sports Medicine and Adult Reconstructive Surgery, Nanjing Drum Tower Hospital, The Affiliated Hospital of Nanjing University Medical School, 321 Zhongshan Road, Nanjing, 210008 Jiangsu PR China; 3 Departments of Laboratory Medicine and Infectious Diseases, Nanjing Drum Tower Hospital, The Affiliated Hospital of Nanjing University Medical School, 321 Zhongshan Road, Nanjing, 210008 Jiangsu PR China

**Keywords:** Post-translational modifications, Monocytes and macrophages

## Abstract

Metformin, traditionally regarded as a hypoglycemic drug, has been studied in other various fields including inflammation. The specific mechanism of metformin’s effect on immune cells remains unclear. Herein, it is verified that LPS-induced macrophages are characterized by enhanced endogenous fatty acid synthesis and the inhibition of fatty acid synthase (FASN) downregulates proinflammatory responses. We further show that metformin could suppress such elevation of FASN as well as proinflammatory activation in macrophages. In vivo, metformin treatment ameliorates dextran sulfate sodium (DSS)-induced colitis through impairing proinflammatory activation of colonic lamina propria mononuclear cells (LPMCs). The reduction of FASN by metformin hinders Akt palmitoylation, which further disturbs Akt membrane attachment and its phosphorylation. Metformin-mediated suppression of FASN/Akt pathway and its downstream MAPK signaling contributes to its anti-inflammatory role in macrophages. From the perspective of immunometabolism, our work points towards metformin utilization as an effective and potential intervention against macrophages-involved inflammatory diseases.

## Introduction

Macrophages display functional plasticity [1]. In recent years, researchers have been recognizing that activation, differentiation, and polarization of macrophages can trigger distinct changes in intracellular metabolic pathways (metabolic reprograming), which in turn contribute to shaping the immune responses and immune cell fate [2, 3]. For example, proinflammatory macrophages possess enhanced glycolysis, whereas IL-4-induced macrophages rely on mitochondrial oxidative phosphorylation (OXPHOS) [4, 5]. Furthermore, several studies have also revealed that proinflammatory stimuli upregulate de novo fatty acid synthesis in macrophages [6]. Though a number of studies have indicated that exogenous fatty acid activates TLR related inflammatory signaling pathway in immune cells [7, 8], the endogenous fatty acid has been being evidenced to regulate inflammation and immune as well [9, 10]. Intracellular palmitic acid synthesized by fatty acid synthase (FASN) can be attached to protein as a modification called palmitoylation [11–14]. Palmitoylation could enhance the hydrophobicity of protein and further influence protein trafficking and functions, which bridges fatty acid metabolism and other cellular activities including inflammatory responses. It has been reported that palmitoylation of MYD88 and NOD participated in regulating inflammatory responses [11, 12, 15]. Warburg effect as a cancer target [16], we inferred that fatty acid synthesis was a novel target pathway to suppress proinflammatory activation of macrophages.

Metformin, as a first-line drug for type 2 diabetes mellitus, reduces blood glucose partly via activating AMP-activated protein kinase (AMPK) [17]. In addition, several investigations have reported that the drug improves liver triglyceride accumulation, dyslipidemia, and atherosclerosis [18–20]. In the past years it has also been suggested that metformin elicits anti-inflammatory action via acting on macrophages directly [21–23]. Owing to the importance of fatty acid synthesis to proinflammatory activation in macrophages as well as the possible effect of metformin on lipid metabolism, we hypothesized that metformin could inhibit proinflammatory activation of macrophages via reducing their fatty acid synthesis, which further alleviated inflammation.

In our study, we show that proinflammatory macrophages are characterized by increased endogenous fatty acid synthesis and the inhibition of FASN downregulates proinflammatory responses of macrophages. We further identify that metformin could suppress LPS-induced elevation of FASN and proinflammatory responses in macrophages as well. The inhibition of FASN by metformin reduces Akt palmitoylation, which is closely associated with downregulated Akt membrane recruitment and phosphorylation. Metformin-mediated suppression of FASN/Akt pathway and its downstream MAPK signaling contributes to its anti-inflammatory role in macrophages. Consistent with in vitro results, metformin treatment ameliorates dextran sulfate sodium (DSS)-induced colitis through impairing proinflammatory activation of colonic lamina propria mononuclear cells (LPMCs).

## Materials and methods

### Cell culture

Murine bone marrow-derived macrophages (BMDMs) were prepared as described previously [24]. Mouse femurs and tibias (8-week old, male) were cut at both ends, and then flushed with 1640 medium (Corning) using a syringe. The bone marrow cells filtered through a 70-μm strainer were plated on sterile petri dishes and incubated for 7 days in 1640 medium containing 10% (vol/vol) heat-inactivated fetal bovine serum (FBS) (Biological Industries), penicillin, streptomycin (Gibco), and 20 ng/ml mouse macrophages colony-stimulating factor (M-CSF) (MCE, HY-P7085). Murine RAW 264.7 macrophages (purchased from the Cell bank of Type Culture Collection of Chinese Academy of Science) were cultured in DMEM medium (WISENT) containing 10% FBS, penicillin, and streptomycin. Macrophages with a 2-h pretreatment of 2 mM metformin or 20 μM C75 were treated with 100 ng/ml LPS (Sigma, L2630) for 30 min or 4 h.

### Transductions of shRNA

Two independent shRNA lentiviral constructs were used for knockdown of mouse *Fasn*. BMDMs (1 × 10^6^ cells per well) or RAW 264.7 macrophages (2 × 10^5^ cells per well) were seeded in six-well plates. Culture medium was replaced with fresh medium containing 10% FBS on the day before transduction. Cells were transduced with shRNA lentiviral constructs against mouse *Fasn* or control shRNA constructs according to the manufacturer’s instructions.

(the targeting sequence of shFASN-1: CCGGCGTCTATACCACTGCTTACTACTCGAGTAGTAAGCAGTGGTATAGACGTTTTTG;

the targeting sequence of shFASN-2: CCGGCCCTTGATGAAGAGGGATCATCTCGAGATGATCCCTCTTCATCAAGGGTTTTTG).

### Overexpression of Akt (wild type or mutant type)

The Akt overexpression plasmids of wild type (WT) and mutant type (C60S) were constructed with the assistance of GeneChem Co., Ltd (Shanghai, China) from CV061 vector (CMV-MCS-3FLAG-SV40-Puromycin). Adherent RAW 264.7 macrophages were transfected with either wild or mutant plasmids, using Lipofectamine^TM^ 3000 reagent (Invitrogen, L3000015). The processes were as the manufacturer’ instructions indicated.

### Free fatty acid measurements

Intracellular free fatty acid content was determined using free fatty acid assay kit (Solarbio, BC0595) as described in the manufacturer’s instructions, the principle of which was that free fatty acid could bind with Cu^2+^ forming copper salt. The copper salt was soluble in chloroform, so it was possible to calculate free fatty acid content when measuring the content of copper solubilized in the organic solvent. Briefly, cell samples were homogenized in lysis buffer provided by the kit. After centrifugation, the supernatant was collected for assaying. The extract was mixed adequately with an organic solvent (n-heptane: methyl alcohol: chloroform = 24:1:25) and CuSO_4_ solution, and then centrifugated to separate the oil phase (upper) from the water phase (lower). The supernatant was pipetted to measure the copper salt, which has bind with free fatty acid. Total protein concentrations were determined using BCA assay (KeyGEN, KGP903) for normalization of free fatty acid content.

### Acly-biotin exchange

Acly-biotin exchange (ABE) assay was based on a previously published protocol [25]. Briefly, samples were homogenized in lysis buffer (150 mM NaCl, 50 mM Tris, 5 mM EDTA, pH 7.4, 1 mM PMSF, protease inhibitor cocktail), which additionally contained 50 mM N-ethylmaleimide (NEM) (Thermo Fisher, 23030). Lysates were sonicated and then incubated overnight at 4 °C with gentle end-over-end rotation. The supernatants obtained by centrifugation at 12,000 rpm for 20 min were subject to chloroform-methanol protein precipitation (supernatant: methanol: chloroform: water, 1:4:1.5:3) to remove excess NEM. The precipitate was dissolved in 4% SDS buffer (4% SDS, 50 mM Tris, 5 mM EDTA, pH 7.4) at room temperature and then divided into two equal portions. One portion was mixed with +HAM buffer (pH 7.4); the other portion was added to -HAM buffer (pH 7.4) correspondingly. +HAM buffer was a solution including 0.7 M hydroxylamine (Sigma, 438227), 1 mM HPDP-biotin (Thermo Fisher, 21341), 0.2% Triton X-100, 1 mM PMSF, and protease inhibitor cocktail, whereas -HAM buffer contained 50 mM Tris, 1 mM HPDP-biotin, 0.2% Triton X-100, 1 mM PMSF and protease inhibitor cocktail. All samples were incubated at room temperature for 1 h with end-over-end rotation, and then subject to chloroform-methanol protein precipitation again. Protein was solubilized in 2% SDS buffer and diluted by 1/20, followed by incubation with streptavidin-agarose (Thermo Fisher, 20347) at room temperature for 90 min. The immunoprecipitated beads were washed three times with lysis buffer and eluted with 2× SDS loading sample buffer.

Immunoprecipitation-ABE (IP-ABE) assay was employed to confirm a palmitoylated protein of interest [26]. The target protein was initially purified by an immunoprecipitation step using an antibody directed against it. The subsequent procedures had a similar principle with ABE assay. Briefly, culture cells were homogenized in lysis buffer containing NEM for 1 h at 4 °C and the supernatant was incubated with the primary antibody overnight at 4 °C. On the second day, the protein A/G agarose was added into the supernatant and the incubation lasted another 2 h. The beads were washed three times with lysis buffer (pH 7.4) and then suspended in hydroxylamine-containing lysis buffer at room temperature for 1 h. The beads were washed three times again and interacted with HPDP-biotin (4 μM, pH 6.4) at 4 °C for another hour. The immunoprecipitated samples were analyzed by western blot using anti-Akt antibody and streptavidin-HRP (Beyotime, A0303).

### Subcellular fractionation

Cell membrane protein and cytoplasmic protein extraction reagent (KeyGEN, KGP3100) was utilized following its instructions. In brief, cells were harvested in lysis buffer containing protease inhibitor cocktail and DTT. The lysates were incubated on ice for 1 min and then vortexed for 30 s, which was repeated five or more times to break up over 90% cells. After centrifugation at 12,000 rpm for 10 min at 4 °C, the obtained supernatants were the cytoplasmic protein, whereas the pellets were subsequently homogenized in extraction buffer to get the membrane protein. All protein was mixed with SDS loading buffer and heated at 95 °C for 10 min.

### Western blotting

Cells or tissues were harvested and homogenized on ice using RIPA lysis buffer (Beyotime, P0013B) containing protease inhibitor cocktail (Roche, 04693132001), phosphatase inhibitor cocktail (Roche, 04906837001) and PMSF (KeyGEN, KGP610). After centrifugation at 12,000 rpm for 20 min at 4 °C, the supernatant concentrations were determined by BCA assay (KeyGEN, KGP903) according to its instructions. Equal amounts of protein were subjected to 8–12% SDS-PAGE gels (BIO-RAD, 79-06-1) and then transferred to polyvinylidene difluoride membranes (Millipore, IPVH00010). The membranes were blocked in tris-buffered saline with 0.1% Tween (TBS-T) containing 5% non-fat milk or bovine serum albumin for 1 h at room temperature, and then incubated with primary antibodies overnight at 4 °C followed by interaction with HRP-linked secondary antibodies on the second day. Specific signaling was generated using chemiluminescent substrate (Share-Bio, sb-wb012) and recorded with a CCD camera (Tanon, 5200 Multi).

### Immunofluorescence

Cells seeded on glass dishes were fixed with 4% paraformaldehyde (vol/vol in PBS) for 30 min. The fixed cells were washed twice with PBS, and then permeabilized using 0.2% Triton X-100 (vol/vol in PBS). Next the cells were blocked with 5% bovine serum albumin (in PBS) for 30 min, followed by incubation with primary antibodies overnight at 4 °C. On the second day, the cells continued to interact with secondary antibodies at room temperature in the dark for 1 h after removing excess primary antibodies. Immunoreactive signaling was visualized with fluorescence microscope (ZEISS).

### RNA isolation and quantitative RT-PCR

Total RNA was isolated from cultured cells or tissues using Trizol reagent (Takara, 9109). Reverse transcription reactions were performed to synthesize cDNA from 1 μg of total RNA using HiScript III Q RT SuperMix (Vazyme, R323-01) as described in the manufacturer’s instructions. A 20 μl mixture containing diluted cDNA, gene-specific primers and ChamQ Universal SYBR qPCR Master Mix (Vazyme, Q711-02) was subjected to quantitative RT-PCR. The temperature and time were set according to the manufacturer’s protocol. The 2-δδCT was calculated to determine the relative mRNA levels of a target gene.

### DSS-induced colitis mouse model

Six-week-old male C57BL/6 mice (purchased from Nanjing Medical University) were housed under specific pathogen-free conditions at Nanjing Drum Tower Hospital. Colitis was induced by adding 2.5% DSS (MP Biomedicals, 02160110) to their drinking water from day 0 to day 8 and 5–10 mice per group were recommended [27]. Mice were randomly divided into five groups, consisting of water group, DSS group, metformin-1 group, metformin-2 group, and C75 group. The latter four groups all drunk water containing DSS. 100 mg/kg metformin hydrochloride (Sigma, 1396309), 200 mg/kg metformin or 5 mg/kg C75 (MCE, HY-12364) in 200 μl saline was daily administered to metformin-1 group, metformin-2 group, or C75 group by intraperitoneal injection, starting from day 3. The dose of these drugs was based on previous studies [28, 29]. Animal care and handling was approved by the Institutional Animal Care and Use Committee of the Affiliated Drum Tower Hospital of Nanjing University Medical School (Nanjing, China), and was performed following the guidelines set by the Animal Care Committee.

### Assessment of disease activity

Weight changes and characteristics of feces were monitored daily throughout the experiment [27]. The extents of weight loss, diarrhea, and fecal bleeding were separately quantitively recorded as a 0–4 score [30]. The scoring criteria were conducted as below. Weight loss: 0 points, <5% weight loss;1 point, 5–10% weight loss; 2 points, 10–15% weight loss; 3 points, 15–20% weight loss; 4 points, >20% weight loss. Stool consistency: 0 points, pellets; 2 points, paste-like/semi-formed stool; 4 points, liquid stool. Fecal bleeding: 0 points, no bleeding; 2 points, slightly bleeding; 4 points, gross bleeding. The disease activity index (DAI) was calculated by combining scores of the three signs above. All mice were euthanized on day 8 and the colons were collected for length measurement, histopathological analysis, LPMCs isolation, etc. Assessment of disease activity (including DAI score, length measurement and histopathological analysis) were conducted by two blinded individuals.

### Immunohistochemistry

Paraffin-embedded colonic sections of mice were deparaffinized using xylene, and rehydrated through graded ethanol. Immunohistochemistry kit (MXB Biotechnologies, KIT-9720) was used according to its manufacturer’s instructions for next processes. Briefly, 3% (vol/vol) H_2_O_2_ in methanol was firstly utilized to block endogenous peroxidase. Then sections immersed in citrate buffer were heated in microwave for 15 min to retrieve antigen. After cooling to room temperature, sections were blocked using goat serum and then incubated with primary antibodies overnight at 4 °C, followed by biotin-conjugated secondary antibodies incubation and subsequent streptavidin-HRP incubation. Immunoreactive tissues were visualized with DAB (MXB Biotechnologies, DAB-0031) as a chromogen and hematoxylin as counterstain.

### Histopathological analysis

Formalin-fixed and paraffin-embedded colon tissues were sectioned 5 μm thick and stained with hematoxylin and eosin (H&E). The histopathological analysis focused on colonic epithelial damage and inflammatory cells infiltration, quantitively with a total score ranging from 0 to 12 [29]. Colonic epithelial damage was described as below: 0 points, normal; 1 point, hyperproliferation, irregular crypts, and goblet cell loss; 2 points, mild to moderate crypt loss (10–50%); 3 points, severe crypt loss (50–90%); 4 points, complete crypt loss but intact surface epithelial; 5 points, small- to medium-sized ulcer (<10 crypt widths); 6 points, large ulcer (≥10 crypt widths). Inflammatory cell infiltration in mucosa, submucosa, and muscle/serosa was assessed separately [mucosa (0 points, normal; 1 point, mild; 2 points, modest; and 3 points, severe); submucosa (0 points, normal; 1 point, mild to modest; and 2 points, severe); muscle/serosa (0 points, normal; and 1 point, mild to severe)]. Scores for epithelial damage and infiltration were added together in order to evaluate the severities from the perspective of histopathology.

### LPMCs isolation

Murine LPMCs were isolated according to a previous protocol published in Nature [31]. In brief, the freshly obtained colons were cut into 4–5 cm long pieces and cleared by flushing colonic cavity with a syringe filled with sterile PBS. Then we opened the colons longitudinally in order to wash them cleaner and exposed the mucosa more thoroughly. Colon pieces were sliced smaller (1 cm long) and incubated in pre-digestion solution containing 5 mM EDTA and 1 mM DTT (Roche, 10197777001) to separate epithelial cells. Finally, the remaining pieces were digested with collagenase D (Roche, 11088858001) under slow rotation and passed through a 40-μm cell strainer to collect LPMCs. The tissues could be digested several times according to the actual situation. LPMCs could be collected for further experiments.

### Antibodies and primers

Anti-β-actin (Sigma, A5441), anti-GAPDH (Cell Signaling Technology, 5174), anti-α-tubulin (Cell Signaling Technology, 2144), anti-Na^+^-K^+^ ATPase (proteintech, 14418-1-AP), anti-ACACA (Cell Signaling Technology, 3662), anti-ACLY (proteintech, 15421-1-AP), anti-FASN (Cell Signaling Technology, 3180), anti-AMPK (Cell Signaling Technology, 2532), anti-p-AMPK (Cell Signaling Technology, 2535), anti-p-ACACA (Cell Signaling Technology, 3661), anti-NF-κB p65 (Cell Signaling Technology, 8242), anti-p-NF-κB p65 (Cell Signaling Technology, 3033), anti-Akt (Cell Signaling Technology, 4691), anti-p-Akt (Cell Signaling Technology, 4060), anti-mTOR (Cell Signaling Technology, 2983), anti-p-mTOR (Cell Signaling Technology, 5536), anti-p38 (Cell Signaling Technology, 8690), anti-p-p38 (Cell Signaling Technology, 4511), anti-ERK (Cell Signaling Technology, 9102), anti-p-ERK (Cell Signaling Technology, 4370), anti-JNK (Cell Signaling Technology, 9252), anti-p-JNK (Cell Signaling Technology, 4306).

*Tnfα* (forward GCCTCTTCTCATTCCTGCTT, reverse TGGGAACTTCTCATCCCTTTG), *Il-1β* (forward TGGCAACTGTTCCTG, reverse GGAAGCAGCCCTTCATCTTT), *Il-6* (forward GCCTTCTTGGGACTGATGCT, reverse TGCCATTGCACAACTCTTTTC), *Nlrp3* (forward ACCAGCCAGAGTGGAATGAC, reverse ACCTGCTTCTCACATGTCGT), *Cox-2* (forward GCCTACTACAAGTGTTTCTTTTTGCA, reverse CATTTTGTTTGATTGTTCACACCAT), *Nos2* (forward AATCTTGGAGCGAGTTGTGG, reverse CAGGAAGTAGGTGAGGGCTTG), *β-actin* (forward GTGACGTTGACATCCGTAAAGA, reverse GCCGGACTCATCGTACTCC).

### Statistical analysis

Statistical analysis was performed using GraphPad Prism 7. All data ware presented as mean ± SD. The difference between the two groups was analyzed by *t*-test (two-tailed, unpaired), while multiple groups were analyzed by one-way ANOVA (with post hoc comparison using Dunnett’s test). Each group represented at least three independent experiments (in vitro) or five animals (in vivo). No samples or animals were excluded from being analyzed. The homogeneity of variance was analyzed by *F* test. *P* < 0.05 were considered statistically significant.

## Results

### The inhibition of FASN downregulates inflammatory responses in macrophages

**Fig. 1:**
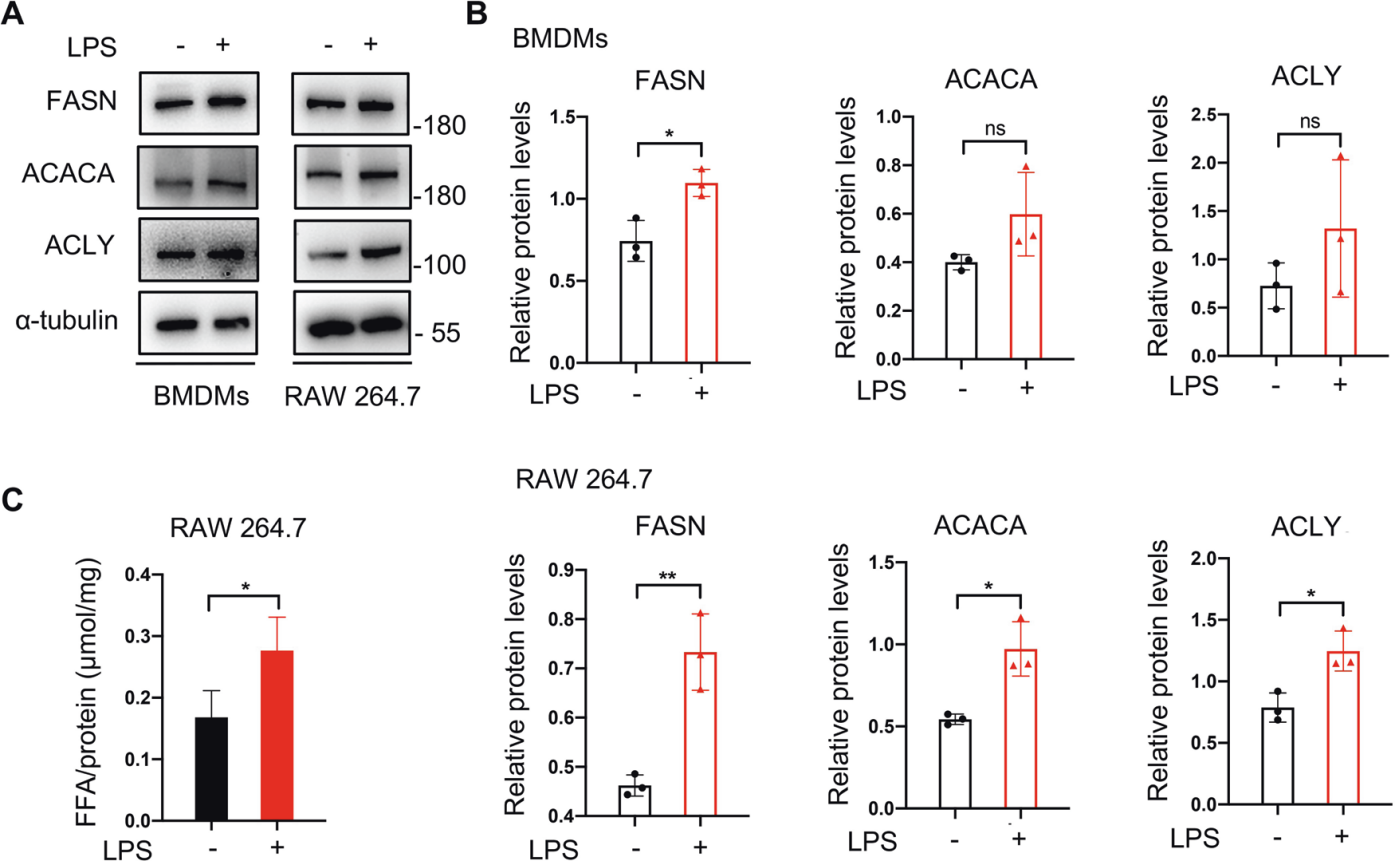
LPS stimuli promoted de novo fatty acid synthesis pathway in macrophages. **A**, **B** Murine bone marrow-derived macrophages (BMDMs) or murine RAW 264.7 macrophages were cultured in the absence or presence of 100 ng/ml lipopolysaccharide (LPS). The protein levels of fatty acid synthase (FASN), acetyl-CoA carboxylase (ACACA), and ATP citrate lyase (ACLY) were determined by Western blotting. Representative images of western blotting (**A**) and a statistical analysis of relative protein levels (**B**). **C** The intracellular free fatty acid (FFA) was determined by free fatty acid assay kit after exposure to LPS (*n* = 4). Data were all represented as means ± SD. *p < 0.05, **p < 0.01, ***p < 0.001, ****p < 0.0001, *t*-test.

**Fig. 2:**
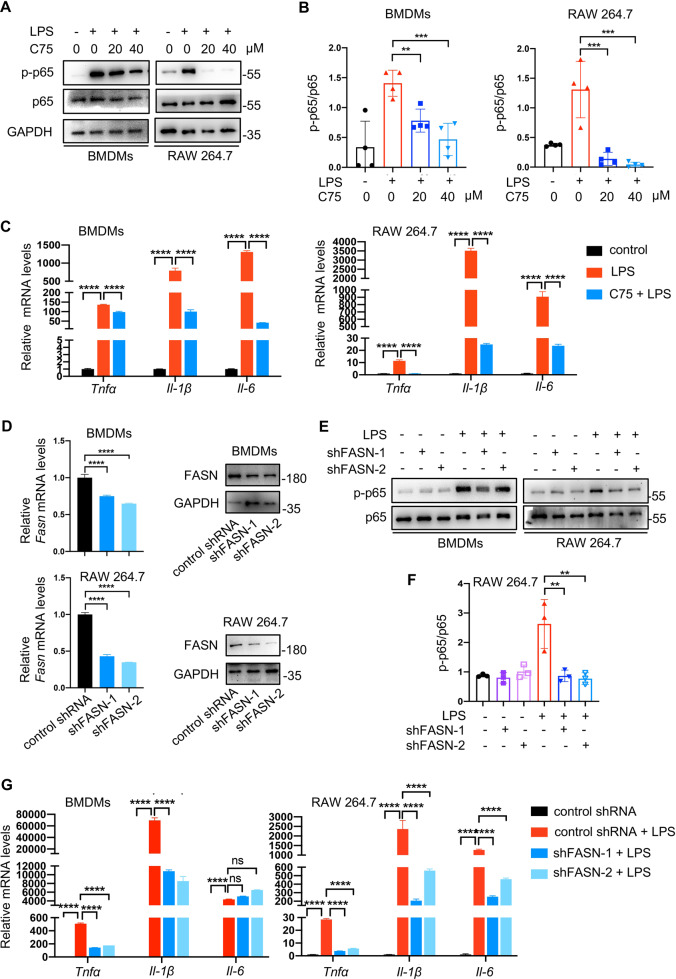
The inhibition of fatty acid synthesis suppressed proinflammation responses of macrophages. **A**, **B** Treatment with various concentrations of C75 (FASN inhibitor) inhibited the phosphorylation of p65 in macrophages exposed to 100 ng/ml LPS for 30 min, as determined by Western blotting. Representative images of western blotting (**A**) and quantification of p-p65/p65 ratios (**B**). **C** The relative mRNA levels of *Tnfα, Il-1β, and Il-6* were detected by qRT-PCR after treatment with 100 ng/ml LPS in the presence or absence of 20 μM C75 for 4 h (*n* = 3). **D** Macrophages were transduced with *Fasn* shRNA or control shRNA lentivirus constructs. The efficiency of knockdown was confirmed by qRT-PCR (*n* = 3, left) and Western blotting (right). **E**, **F** Knockdown of *Fasn* suppressed p65 phosphorylation in macrophages triggered by 100 ng/ml LPS for 30 min, as determined by Western blotting. Representative images of western blotting (**E**) and quantification of p-p65/p65 ratios (**F**). **G** *Fasn*-knockdown macrophages were treated with 100 ng/ml LPS for 4 h. The mRNA levels of *Tnfα, Il-1β,* and *Il-6* were analyzed by qRT-PCR (*n* = 3). Data were represented as means ± SD. *p < 0.05, **p < 0.01, ***p < 0.001, ****p < 0.0001, ANOVA.

To vertify the relationship between de novo fatty acid synthesis and inflammatory responses in macrophages, we established an inflammation model of LPS-triggered macrophages. De novo fatty acid synthesis is regulated by three key lipogenic enzymes, FASN, acetyl-CoA carboxylase (ACACA), and ATP citrate lyase (ACLY) [32], so we firstly detected their protein levels to analyze whether LPS stimulus influenced fatty acid synthesis in macrophages. Indeed, there was an elevation of these mentioned enzymes in macrophages after exposure to LPS (Fig. 1A, B). Furthermore, intracellular free fatty acid also increased (Fig. 1C). As FASN converting acetyl-CoA and malonyl-CoA into fatty acid (mainly palmitic acid) works in the terminal catalytic step [32], we focused on it and evaluated its influence on macrophage activation through using a FASN inhibitor C75 or shRNA lentiviral constructs against *Fasn*. C75 treatment downregulated LPS-induced p65 phosphorylation (Fig. 2A, B) as well as the enhanced *Tnfα, Il-1β*, and *Il-6* levels (Fig. 2C, Fig. S1-A). These molecules are typical proinflammatory transcriptional factor and cytokines [33, 34]. In addition, we transduced macrophages with shRNA lentiviral constructs to rule out the off-target influence of C75 (Fig. 2D). Knockdown of FASN got a similar result to C75 utilization (Fig. 2E–G). Taken together, these results suggested that proinflammatory activation of macrophages depended on enhanced fatty acid synthesis.

### Metformin alleviates inflammation through inhibiting FASN/Akt/p65 pathway in macrophages

**Fig. 3:**
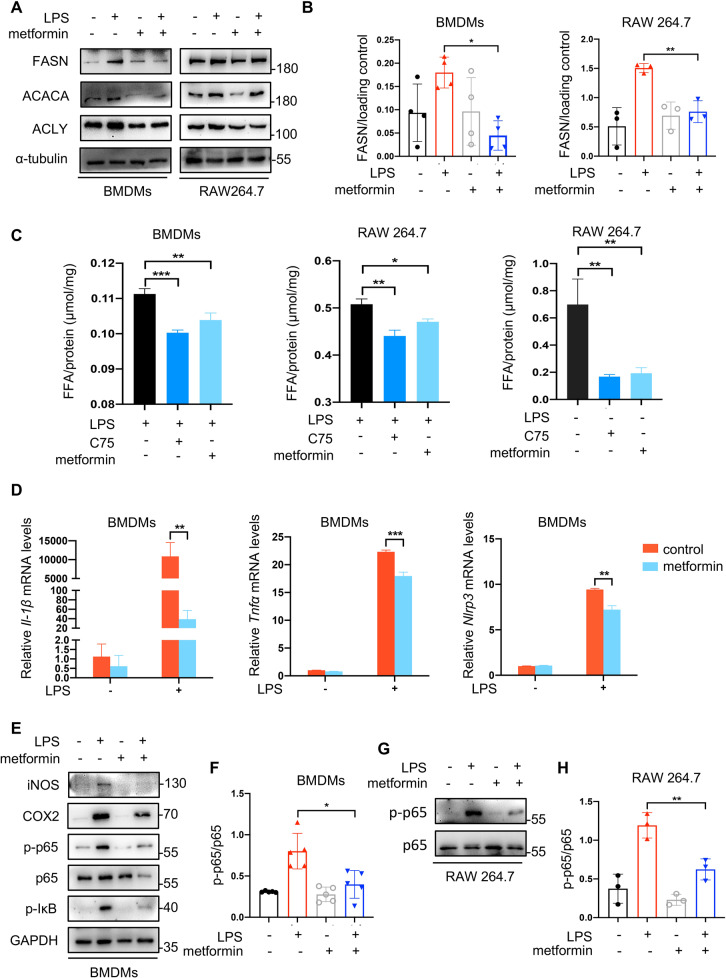
Metformin had an inhibitory effect on fatty acid synthesis as well as proinflammatory responses of macrophages. **A**, **B** Macrophages were treated with 100 ng/ml LPS in the presence or absence of 2 mM metformin. The protein levels of FASN, ACACA, and ACLY were determined by Western blotting. Representative images of western blotting (**A**) and a statistical analysis of relative FASN levels (**B**). **C** Intracellular free fatty acid (FFA) was detected by free fatty acid assay kit (*n* = 6). **D** The mRNA levels of *Il-1β, Tnfα*, and *Nlrp3* in metformin-treated BMDMs were determined by qRT-PCR after 4-h treatment (*n* = 3). **E**–**H** The proinflammatory protein levels of macrophages were assayed by Western blotting. Representative images of western blotting (**E**, **G**) and quantitative analyses (**F**, **H**). Data were all represented as means ± SD. *p < 0.05, **p < 0.01, ***p < 0.001, ****p < 0.0001, ANOVA or *t*-test.

Since metformin has been reported to suppress fatty acid accumulation in liver cells [35], we predicted metformin had a similar effect on fatty acid synthesis in macrophages. Indeed, we observed the expression of FASN, ACACA, and ACLY was lower in metformin-treated proinflammatory macrophages than untreated ones (Fig. 3A, B). In addition, metformin treatment, similar to C75, decreased intracellular free fatty acid content in macrophages as well (Fig. 3C). Next, we continued to identify the influence of metformin on macrophage proinflammatory responses. It was found that metformin inhibited LPS-induced enhancement of proinflammatory cytokines (Fig. 3D, Fig. S1-B). The inflammation-associated protein levels of iNOS, COX2, p-p65, and p-IκB also followed a downward trend when macrophages being exposed to metformin (Fig. 3E–H).

Indeed, many articles have claimed that metformin suppresses proinflammatory responses of macrophage through activating AMPK [21]. Therefore, we preliminarily detected the phosphorylation levels of AMPK in macrophages. Consistently, our results also showed that AMPK phosphorylation in macrophages exposed to metformin was enhanced (Fig. S1-C). As our aim was to explain the anti-inflammatory mechanism of metformin from the immunometabolism perspective, we explored other possible signaling pathways instead of the classical one (AMPK).

**Fig. 4:**
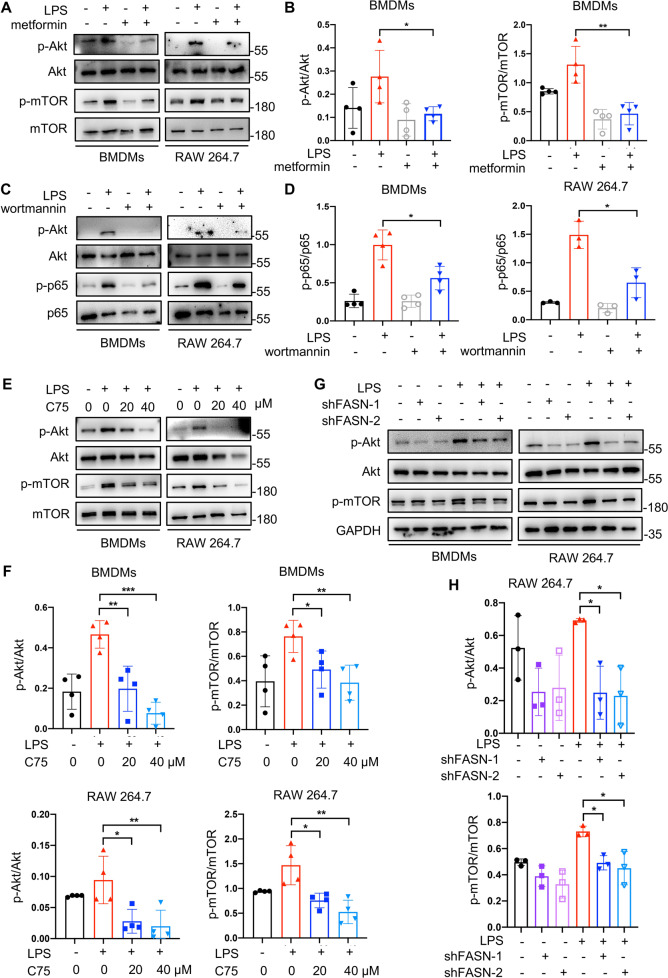
Metformin alleviated inflammation through inhibiting FASN/Akt/p65 pathway in macrophages. **A**, **B** The activation of p-Akt/p-mTOR were determined by western blotting after macrophages were stimulated with 100 ng/ml LPS or LPS plus 2 mM metformin. Representative images of western blotting (**A**) and a statistical analysis of p-Akt/Akt and p-mTOR/mTOR ratios (**B**). **C**, **D** When 20 nM wortmannin (an inhibitor of PI3K/Akt pathway) was used to hinder the activation of Akt, LPS-induced p65 phosphorylation was reduced in macrophages as determined by Western blotting. Representative images of western blotting (**C**) and a statistical analysis of p-p65/p65 ratios (**D**). **E**, **F** Macrophages were treated with different concentrations of C75 and then Akt phosphorylation was analyzed. Representative images of western blotting (**E**) and quantification of p-Akt/Akt and p-mTOR/mTOR ratios (**F**). **G**, **H** Macrophages transduced with *Fasn* shRNA lentivirus were triggered by 100 ng/ml LPS for 30 min. Akt phosphorylation was analyzed to confirm the regulative effect of FASN on Akt. Representative images of western blotting (**G**) and quantification of p-Akt/Akt and p-mTOR/mTOR ratios (**H**). Data were all represented as means ± SD. *p < 0.05, **p < 0.01, ***p < 0.001, ****p < 0.0001, ANOVA or *t*-test.

Combined with the previous data (Figs. 1–3), it was indicated that metformin might alleviate inflammation through suppressing intracellular fatty acid synthesis in macrophages. We further assessed the downstream mechanisms of metformin, and in other words, the downstream pathways affected by fatty acid synthesis. Akt, as a kinase involved in a series of signaling pathways, influences various cell functions and fates, exactly including metabolism and inflammatory responses. In addition, the Akt/mTOR pathway exactly had a cross talk with AMPK [36]. Therefore, we analyzed the Akt/mTOR pathway in macrophages. Indeed, phosphorylation of Akt (Ser 473) and mTOR was elevated in LPS-stimulated macrophages; however, both of them were downregulated under the influence of metformin (Fig. 4A, B; Fig. S1-D). To further investigate whether metformin-induced inhibition of p-Akt was related to its anti-inflammatory role in macrophages, we chose a PI3K inhibitor, wortmannin, to inhibit the phosphorylation of Akt and observed the following changes of macrophage activation. p65 was less phosphorylated in macrophages treated with wortmannin (Fig. 4C, D), which suggested that Akt phosphorylation played a crucial role in mediating macrophage activation. Based on the previous reports that Akt activation could be modulated by FASN in cancer cells [37, 38], we were curious whether FASN was as well responsible for the phosphorylation of Akt in macrophages. With pharmacological inhibition (C75) or knockdown of FASN (*Fasn* shRNA lentivirus), LPS-induced p-Akt/p-mTOR pathway was significantly downregulated in macrophages (Fig. 4E–H). Consequently, metformin exerted anti-inflammatory effect on macrophages through inhibiting FASN/Akt pathway.

### Metformin inhibited Akt activation through suppressing FASN-dependent palmitoylation and its mediated membrane recruitment

**Fig. 5:**
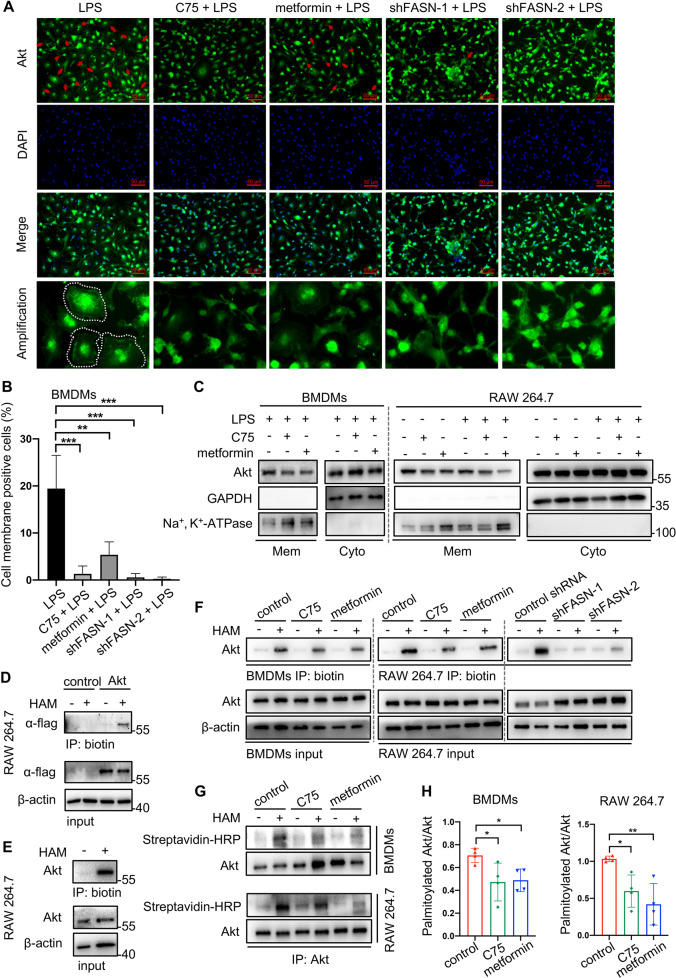
Metformin and FASN regulated the membrane attachment of Akt and its palmitoylation. Macrophages in the presence of LPS were treated with metformin, C75 or *Fasn* shRNA lentivirus. **A**–**C** The membrane localization of Akt in response to a 15-min LPS treatment was determined by immunofluorescence (**A**–**B**) and Western blotting (**C**). **D**, **E** Akt possessed a posttranslational modification called palmitoylation, as determined in RAW 264.7 by acly-biotin exchange (ABE) method. The palmitoylation of exogenously-overexpressed Akt (**D**) and endogenous Akt (**E**). **F**–**H** The effect of metformin and FASN inhibition on Akt palmitoylation was determined by ABE method (**F**) and then confirmed again by immunoprecipitation (IP)-ABE method (**G**). The ratio of palmitoylated Akt/Akt was quantified (**H**). Data were represented as means ± SD. *p < 0.05, **p < 0.01, ***p < 0.001, ****p < 0.0001, ANOVA.

Activated PI3K converts PIP2 to PIP3, which recruits Akt to the plasma membrane [39]. It is precisely on the plasma membrane where two protein kinases, mTORC2 and PDK1, phosphorylate Akt. Membrane attachment is indispensable for the activation of Akt. Traditionally, pleckstrin homology (PH) domain of Akt is responsible for binding to PIP3 [40, 41]. The results above showed that metformin and FASN inhibition suppressed Akt phosphorylation. In this part, we continued to uncover that Akt membrane localization was actually disturbed by metformin as well as FASN inhibition, the same as the reduction of Akt phosphorylation (Fig. 5A–C). Palmitic acid can be converted to palmitoyl-CoA and covalently attached to cysteine residues of peptide chains via thioester linkage, which is called *S*-palmitoylation. Palmitoylation is believed to facilitate membrane localization of protein due to the hydrophobicity of the palmitoyl group [13, 14]. It is unclear whether palmitoylation facilitates Akt attaching to membranes or not. The software GPS-Palm is able to calculate whether a protein could be palmitoylated and the possible modified amino acid cites [42]. With GPS-Palm, we predicted that Akt was likely to undergo palmitoylation (Fig. S2). Consistently, no matter the endogenous Akt or exogenous Akt overexpressed by flag-Akt plasmids, Akt palmitoylation was both detected according to a classical method (ABE assay) (Fig. 5D, E). Due to what had been discovered above, we hypothesized that the suppression of FASN caused by metformin contributed to reducing Akt palmitoylation, which further destroyed membrane recruitment and activation (phosphorylation) of Akt. Macrophages treated with C75 or *Fasn* shRNA lentivirus were regarded as a positive control group. The ABE assay showed that metformin indeed cut down endogenous Akt palmitoylation, which was validated again by IP-ABE assay (Fig. 5F–H).

**Fig. 6:**
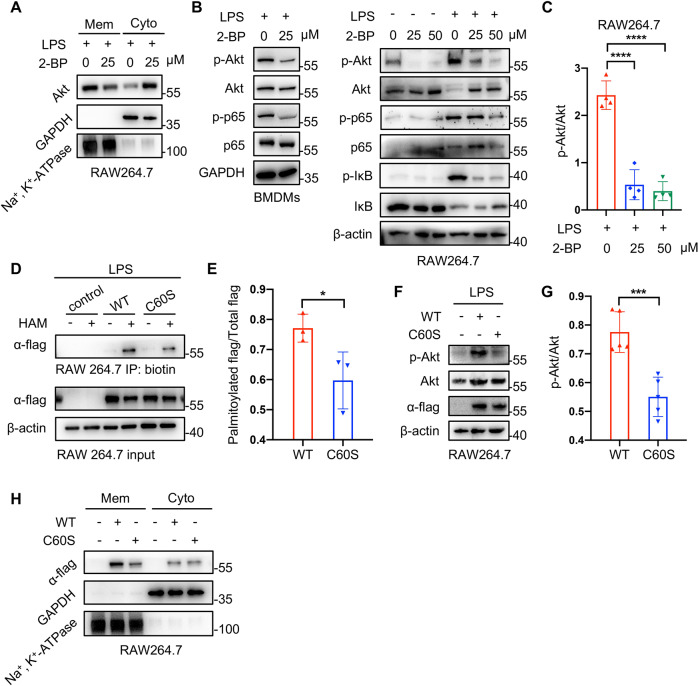
The palmitoylation of Akt promoted its membrane distribution and phosphorylation (activation). **A** 2-bromopalmitate (2-BP, an inhibitor of palmitoylation) reduced the membrane localization of Akt in macrophages. **B**, **C** The activation of Akt was as well suppressed by 2-BP in macrophages. Representative images of western blotting (**B**) and quantification of a p-Akt/Akt ratio (**C**). **D**, **E** RAW 264.7 macrophages were transfected with flag-Akt plasmids to detect the palmitoylated site of Akt. The palmitoylated levels of mutant Akt (C60S) were lower than those of the wild type. Representative images of western blotting (**D**) and quantitative ratios of palmitoylated flag/total flag (**E**). **F**, **G** The mutant Akt (C60S) was less phosphorylated than the wild type. Representative images of western blotting (**F**) and quantitative ratios of p-Akt/Akt (**G**). **H** The mutant Akt (C60S) was less recruited to membrane than the wild type. Data were represented as means ± SD. *p < 0.05, **p < 0.01, ***p < 0.001, ****p < 0.0001, ANOVA or *t*-test.

To further investigate the role of palmitoylation in Akt activation, 2-bromopalmitate (2-BP) (Sigma, 21604), an inhibitor of palmitoylation, was utilized to decrease Akt palmitoylation. As we speculated, inhibiting palmitoylation suppressed the membrane distribution of Akt, as well as the phosphorylation of Akt, p65, and IκB in macrophages (Fig. 6A–C). These results indicated that Akt palmitoylation promoted it to be recruited to membrane, and further be phosphorylated. In addition to the possibility of Akt palmitoylation, the software GPS-Palm (Fig. S2) also predicted the probably modified cysteines of Akt, among which C60 (the sixtieth cysteine) got the highest score. To verify the prediction above and further confirm the importance of Akt palmitoylation to its activation, we constructed two types of flag-Akt plasmids, wild type and mutant type (C60S). As Fig. 6D, E exhibited, Akt was less palmitoylated when the sixtieth cysteine (C60) was mutated, which not only verified C60 was one of the palmitoylated sites, but also implied there existed other cysteines able to be palmitoylated as C60 mutant Akt still had a low level of palmitoylation. Apart from the palmitoylation level, the phosphorylation and membrane attachment of C60 mutant Akt were as well inhibited, compared with the wild type (Fig. 6F–H). These data complemented the above indication that Akt palmitoylation promoted it to be recruited to membrane and further be phosphorylated. Taken together, metformin could inhibit Akt activation through suppressing FASN-dependent palmitoylation and its mediated membrane recruitment.

### MAPK pathways are downstream signaling of FASN and Akt

To further understand the anti-inflammatory mechanism of metformin, we additionally analyzed the MAPK pathways in macrophages (Fig. S3), which have been identified involved in proinflammatory responses [43]. We firstly validated that metformin significantly suppressed LPS-induced phosphorylation of ERK and JNK (Fig. S3-A). Next, in order to reveal the functional effect of FASN on the regulation of MAPK pathways, we investigated whether knockdown of FASN by *Fasn* shRNA transduction could reduce the activation of MAPK pathways. The LPS-stimulated phosphorylation of MAPK was indeed downregulated in macrophages transduced with *Fasn* shRNA, in comparation with control shRNA transduced cells (Fig. S3-B). Similarly, the inhibition of FASN activity by C75 decreased the phosphorylation of p38, ERK, and JNK (Fig. S3-C). Lastly, we wondered whether Akt, as a downstream molecule of FASN, acted on the upstream of MAPK pathways. While using wortmannin to inhibit Akt activation, we observed that inhibition of Akt activation suppressed the phosphorylation of ERK and JNK in LPS-stimulated macrophages (Fig. S3-D). Consequently, it was suggested that MAPK pathways were downstream signaling of FASN/Akt and inhibited by metformin under inflammatory conditions.

### Metformin and C75 ameliorates DSS-induced colitis in mice

**Fig. 7:**
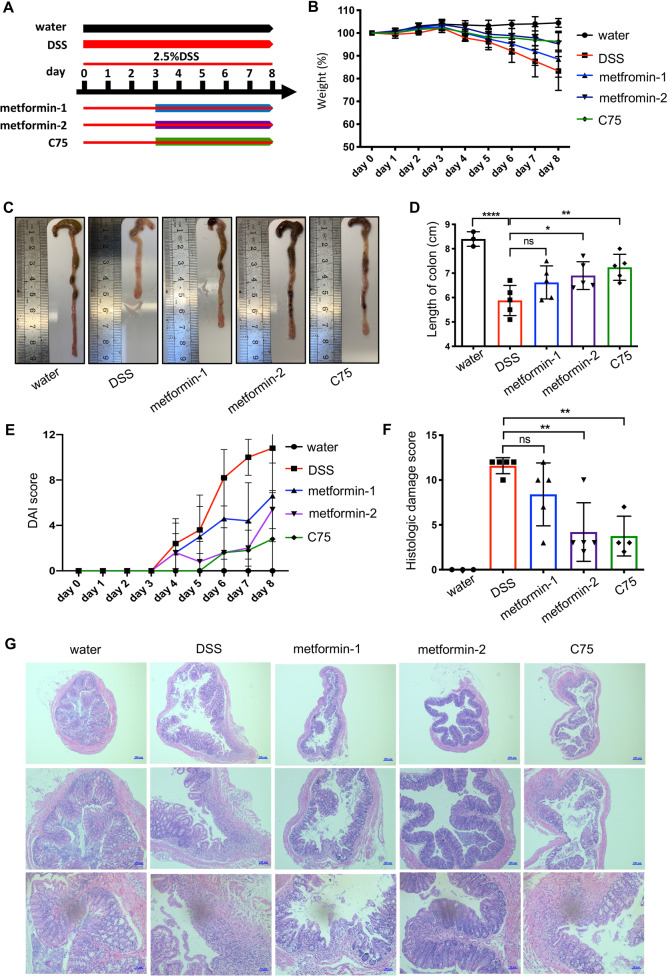
The therapeutic effect of metformin and C75 on dextran sulfate sodium (DSS)-induced colitis mice. **A** An overview of animal experiments (five mice in each group). **B** Weight loss was measured each day during the whole experiment. **C**, **D** After mice were sacrificed, the length of colons was recorded and quantified. **E** Disease activity index (DAI) was calculated according to weight loss and characteristics of feces. **F**, **G** Hematoxylin and eosin (H&E) staining was utilized to evaluate colon damages. The extent of damages was quantified by a score ranging from 0 to 12 (**F**). Representative images of H&E staining (**G**). metformin-1: 100 mg/kg; metformin-2: 200 mg/kg; C75: 5 mg/kg. Data were represented as means ± SD. *p < 0.05, **p < 0.01, ***p < 0.001, ****p < 0.0001, ANOVA.

Inflammatory bowel disease (IBD) is a chronic, incurable disease of the digestive tract, including Crohn’s disease and Ulcerative colitis. An increasing number of studies have found that an abundance of proinflammatory macrophages in inflammatory sites contributes to IBD pathogenesis. Therefore, we evaluated the therapeutic effect of metformin on colitis. As DSS-induced colitis is a chemical damage associated inflammatory process, where adaptive immune is dispensable to colitis progression, this model is suitable for studying innate immune mechanisms and the influence of metformin on innate immune cells in vivo [44]. Weight loss, DAI, and shortening of colon length are general indicators to reflect the severity of DSS-induced colitis [27]. Animal experiment was conducted as Fig. 7A. Colitis mice given metformin or C75 showed significantly diminished weight loss as compared with non-treated colitis mice (Fig. 7B). Consistently, the colon from colitis mice was shorter with a smaller cecum and less stool retained inside comparing to the one from the non-colitis mice (Fig. 7C, D). However, metformin or C75 treatment recovered the colon to their normal condition, nearly the same to the non-colitis one (Fig. 7C, D). We next calculated the DAI scores. The treated groups obtained a lower DAI score than non-treated group (Fig. 7E). In order to analyze the colonic inflammation accurately, histopathological evaluation was conducted on the proximal portions of colons. Large ulceration and massive infiltration of inflammatory cells were evident in colon tissues exposed to DSS (Fig. 7F, G). Conversely, colitis colons with metformin or C75 had markedly attenuated colonic epithelial damage and inflammatory infiltration, indicating their effectiveness on more than clinical parameters (Fig. 7F, G). The quantitative scoring of the histological analysis showed that the mice administrated with 200 mg/kg metformin got a significantly lower score than the colitis mice (Fig. 7F). Therefore, these improvements directly showed that metformin or inhibition of FASN ameliorated macrophages-involved colitis.

### Metformin suppresses proinflammatory responses of colonic LPMCs in DSS-induced colitis mice

**Fig. 8:**
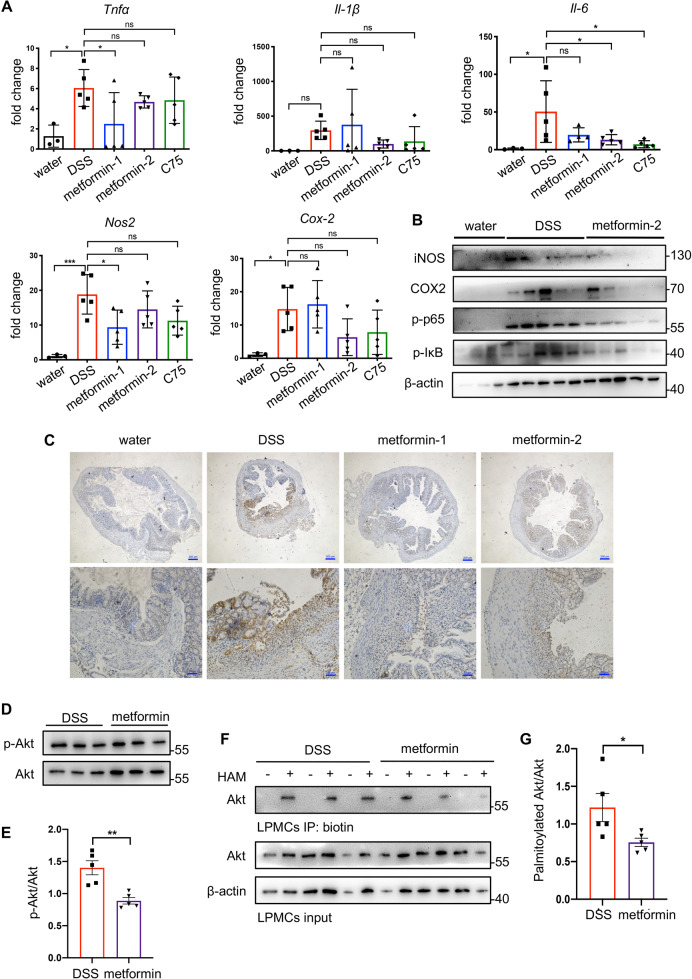
Metformin suppressed proinflammatory responses of colonic LPMCs in DSS-induced colitis mice. Murine lamina propria mononuclear cells (LPMCs) were isolated from colons on day 8. **A** The mRNA levels of *Tnfα*, *Il-1β*, *Il-6*, *Cox-2*, and *Nos2* in LPMCs were determined by qRT-PCR. **B** Proinflammatory proteins in LPMCs were determined by Western blotting. **C** The expression of TNFα in colons was detected by immunohistochemistry. **D**, **E** The phosphorylation of Akt was inhibited by metformin in colonic LPMCs. Representative images of western blotting (**D**) and quantification of p-Akt/Akt ratios (**E**). **F**, **G** The palmitoylation of Akt in colonic LPMCs was detected by the ABE method. Representative images of western blotting (**F**) and quantification of palmitoylated Akt/Akt ratios (**G**). Data were represented as means ± SD. * *p* < 0.05, **p < 0.01, ***p < 0.001, ****p < 0.0001, ANOVA or *t*-test.

We isolated LPMCs from the total colon to demonstrate whether the metformin-induced attenuation of colonic inflammation was, at least partly, attributed to its effective inhibition on colonic macrophages. Similar to in vitro experiments, intracellular free fatty acid of LPMCs seemed to be downregulated by metformin treatment, though without statistical significance (Fig. S4). Since *Tnfα*, *Il-1β*, *Il-6*, *Cox-2*, and *Nos2* were crucial proinflammatory molecules involved in the pathogenesis of IBD, we measured their expressions of LPMCs. Their mRNA levels significantly increased after DSS exposure; but on the contrary, the administration of metformin or C75 decreased the elevation (Fig. 8A). Furthermore, metformin restrained activation of the IκB/p65 pathway in LPMCs (Fig. 8B). The elevated protein levels of COX2 and iNOS in LPMCs of colitis mice were disrupted when receiving metformin administration (Fig. 8B). In line with the above results derived from LPMCs, IHC of colon slices exhibited that colitis mice receiving metformin had lower levels of TNFα in colons (Fig. 8C). To further evaluate the changes of Akt activation and palmitoylation under the influence of metformin in vivo, we detected both levels in LPMCs (Fig. 8D–G). In consistence with in vitro results, metformin utilization reduced Akt phosphorylation (Fig. 8D, E) as well as Akt palmitoylation (Fig. 8F, G).

## Discussion

Numerous studies on the relationship between fatty acid and inflammation have focused on obesity associated diseases, such as atherosclerosis, diabetes, and non-alcoholic fatty liver disease. They have revealed that immune cells can be activated by exogenous fatty acid derived from blood, adipocytes or hepatocytes [7, 8]. However, the contribution of endogenously synthesized fatty acid in immune cells to inflammatory responses remains a limited understanding. Most adult tissues and cells express low levels of FASN and generally obtain free fatty acid from the diet uptake [45]. Nonetheless, higher levels of FASN have been detected in various types of cancer and demonstrated a correlation with malignant degree, which implies probable dysregulation of FASN in some pathological conditions [46–49].

As immunometabolism has been extensively investigated in immune cells, emerging evidence suggests that FASN-generated endogenous fatty acid is an important regulator of immune responses and immune cell fate [2, 6]. T helper 17 (TH17) cells depend on endogenous fatty acid synthesis to produce phospholipids for cellular membranes [50, 51]. FASN-mediated de novo fatty acid synthesis affects the retention of plasma membrane cholesterol and Rho GTPase trafficking, which is important for adhesion, migration and activation of macrophages in diabetes [10]. Fatty acid synthesis is also required for macrophage colony-stimulating factor (M-CSF)-induced differentiation of human monocytes [9]. The mitochondrial uncoupling protein-2 (UCP2) promotes NLRP3 inflammasome activation through upregulating FASN and its mediated fatty acid synthesis [52]. In line with these discoveries, we observed that enhanced expression of FASN was indispensable for LPS-induced proinflammatory responses in macrophages.

To be more detailed, we identified that the LPS-activated Akt/mTOR pathway was hindered by metformin or FASN inhibition in macrophages. It has been well known that membrane recruitment is a major requirement for Akt activation [40]. Since Akt primarily exists in the cytosol, there undoubtedly occurs some unclear molecule events which assist it to approach to plasma membrane. The immunofluorescence and western blotting experiments exhibited that metformin as well as FASN inhibition disrupted the membrane localization of Akt, which guided us to further investigate the probable mechanism of Akt trafficking. A previous report identified that TRAF6, as a E3 ligase promoting Akt ubiquitinated, is crucial for IGF-1-induced Akt membrane distribution and phosphorylation at T308 and S473 in MEFs [53]. With K63-ubiquitination contributing to Akt trafficking, we were interested in the correlation between its post-translational modification and membrane localization. Palmitic acid synthesized by FASN can participate in a protein post-translational modification, called *S*-palmitoylation. *S*-palmitoylation belongs to lipidation [13, 14]. De novo fatty acid synthesis has been validated to contribute to myeloid differentiation primary response protein (MYD88) palmitoylation, which is a major requirement for IRAK4 binding and downstream signal activation [11]. In addition, we were inspired by another fact that palmitoylation-mediated hydrophobicity facilitates protein attaching to membranes. In our study, we identified a previously undescribed *S*-palmitoylated protein, Akt, whose palmitoylation was dependent on FASN. No matter knockdown or pharmacological inhibition of FASN, Akt palmitoylation was reduced. Further, we connected the downregulated palmitoylated Akt with its suppressed membrane localization through confirming the influence of a palmitoyltransferase inhibitor, 2-BP, on Akt distribution and activation. Consequently, the specific mechanism of how metformin regulated Akt activation was to modulate FASN-dependent palmitoylation of Akt.

It is generally recognized that Akt as an upstream regulator increases activity of the transcription factor SREBP-1, resulting in an elevation in FASN mRNA expression [54]. In normal physiological conditions, insulin-responsive cells exposed to insulin, such as adipocytes and hepatocytes, undergo metabolism changes. The activated insulin receptor initiates PI3K/Akt signaling, which further facilitates glycogen and fatty acid synthesis through regulating GSK3β and mTORC1 [55–57]. On the other hand, a number of cancer-associated reports shows that PI3K signaling modulates FASN levels as well in cancerous cells [58–60]. Conversely, it is also verified that FASN acts upstream of Akt and inhibition of FASN downregulates phosphorylated Akt, though the specific mechanism remains unclear [37, 38]. Indeed, our study provided some evidence on the specific mechanism that FASN as a metabolic enzyme regulated palmitoylation-dependent activation of Akt. On the basis of positive self-amplifying feedback between Akt activation and FASN, we speculated that it was meaningful and effective to break the abnormally hyperactive feedback cycle in pathological conditions.

Numerous studies confirmed that metformin had a direct inhibitory effect on proinflammatory responses of macrophages, generally through AMPK activation or inhibiting mitochondrial reactive oxygen species (ROS) production [21–23]. The immunomodulatory properties of metformin have also been suggested to underlie some of its beneficial effects on autoimmune inflammatory diseases [30, 61, 62]. Though metformin is well known as a popular drug regulating cellular metabolism, the contribution of metformin-induced changes in cellular metabolism to its anti-inflammatory function remains unknown. It was significant for our work to be the first to demonstrate that the anti-inflammatory effect of metformin was, at least partly, attributed to inhibiting fatty acid synthesis metabolism in macrophages. Similar with C75, metformin reduced fatty acid synthesis and compromised Akt palmitoylation in LPS-stimulated macrophages, further disrupting its membrane recruitment and phosphorylation. Inactivation of Akt resulted in less phosphorylation of p65 in the end. In brief, we succeeded in uncovering the anti-inflammatory mechanism of metformin from the immune metabolism perspective.

*S-*palmitoylation reactions are directly catalyzed by a family of aspartate-histidine-histidine-cysteine palmitoyl acyl transferases (DHHC-PATs) [14]. Therefore, we plan to further investigate the exact DHHC-PATs mediating Akt palmitoylation in a subsequent study.

## Conclusions

Collectively, it is demonstrated that metformin alleviates inflammation through inhibiting endogenous fatty acid synthesis and its related Akt palmitoylation in macrophages. Our data provides substantial evidence supporting FASN as a therapeutic target and metformin as a potential treatment for macrophages-mediated inflammatory diseases.

## Supplementary information

supplementary figure legend

Figure S1

Figure S2-4

## Data Availability

All data are available in the paper or supplementary materials.
