# Supplementary material for: Metformin alleviates inflammation through suppressing FASN-dependent palmitoylation of Akt
Source: Cell Death Dis. 2021 Oct 12;12(10):934. doi: 10.1038/s41419-021-04235-0 (PMC8511025; doi:10.1038/s41419-021-04235-0)
Supplement: Supplementary file 1 — supplementary figure legend [file 41419_2021_4235_MOESM1_ESM.docx]

**Figure legends of supplementary figures**

Figure S1: (A) BMDMs were treated with 20 μM C75 in the presence or absence of 100 ng/ml LPS treatment for 24 hours. The cytokines were detected by Elisa. (B) BMDMs were treated with 2mM metformin in the presence or absence of 100 ng/ml LPS treatment for 24 hours. The cytokines were detected by Elisa. (C) The phosphorylation of AMP-activated protein kinase (AMPK) was detected by western blotting. (D) Quantification of p-Akt/Akt and p-mTOR/mTOR in RAW 264.7. Data were represented as means ± SD. ** p* < 0.05, *** p* < 0.01, **** p* < 0.001, ***** p* < 0.0001, t-test.

Figure S2: The software GPS-Palm predicted that Akt was likely to undergo palmitoylation.

Figure S3: MAPK pathway was downstream signaling of FASN and Akt. (A) BMDMs were treated with 2mM metformin in the presence or absence of 100 ng/ml LPS treatment for 30 minutes. (B) BMDMs transfected by FASN shRNA lentivirus were triggered by 100 ng/ml LPS for 30 minutes. (C) BMDMs were treated with various concentrations of C75 in the presence or absence of 100 ng/ml LPS for 30 minutes. (D) BMDMs were treated with 20 nM wortmannin in the presence or absence of 100 ng/ml LPS for 30 minutes. The phosphorylation of MAPKs was determined by Western blotting.

Figure S4: The intracellular free fatty acid contents of LPMCs.
