# Supplementary figures and images for: Metformin alleviates inflammation through suppressing FASN-dependent palmitoylation of Akt
Source: Cell Death Dis. 2021 Oct 12;12(10):934. doi: 10.1038/s41419-021-04235-0 (PMC8511025; doi:10.1038/s41419-021-04235-0)

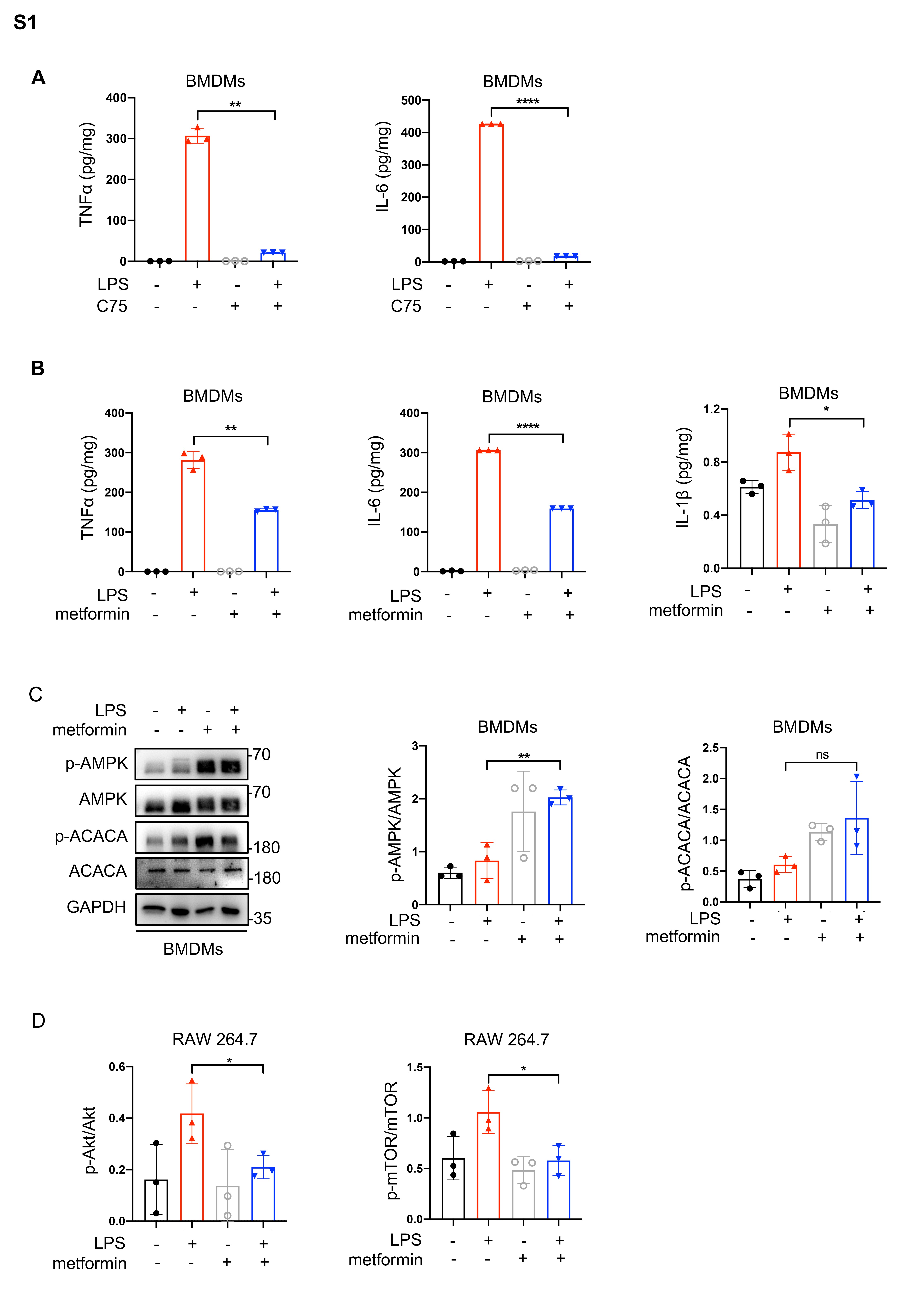

Supplement: Supplementary file 2 — Figure S1 [file 41419_2021_4235_MOESM2_ESM.jpg]

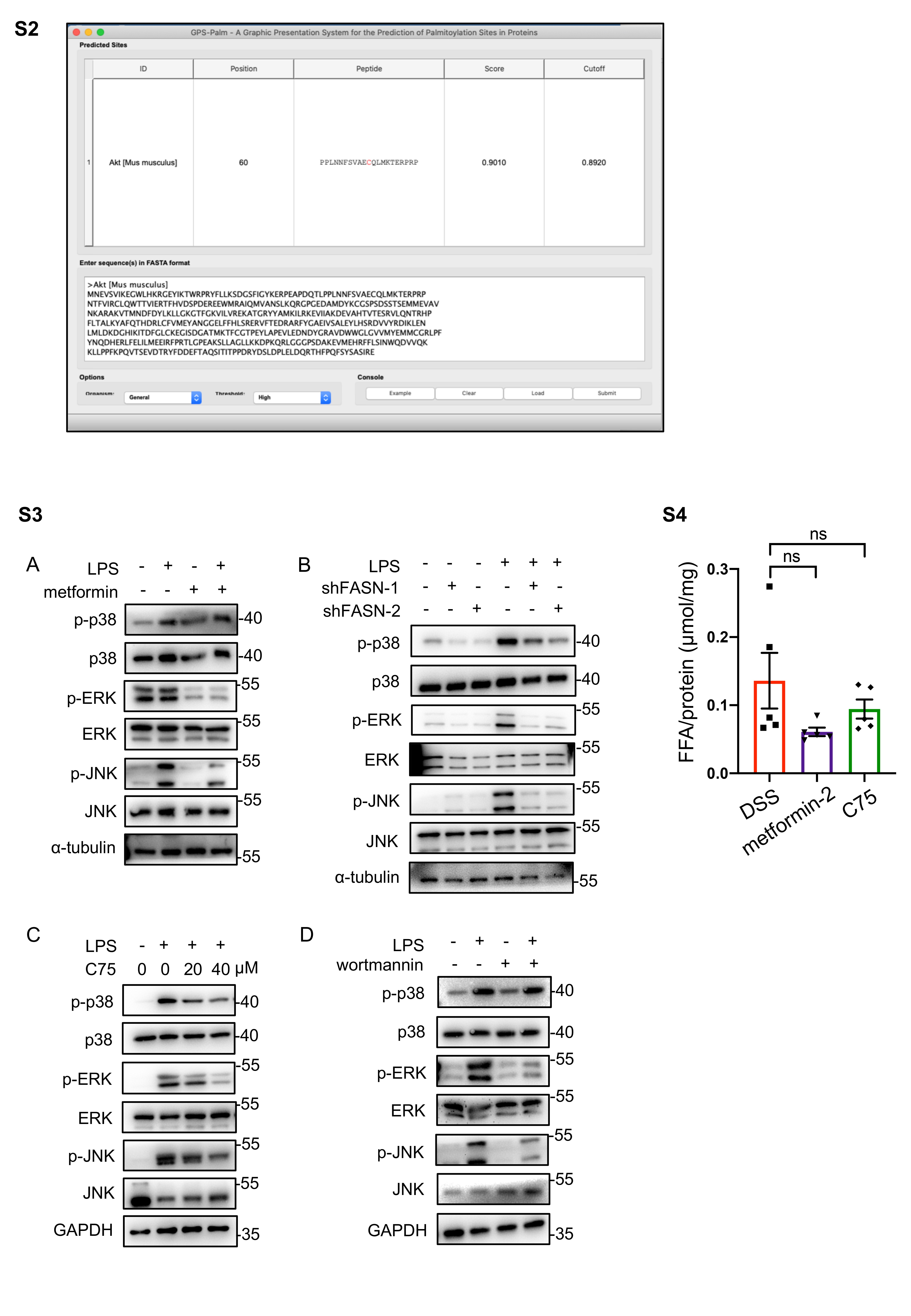

Supplement: Supplementary file 3 — Figure S2-4 [file 41419_2021_4235_MOESM3_ESM.jpg]
